# Exploring the impact of HDL and LMNA gene expression on immunotherapy outcomes in NSCLC: a comprehensive analysis using clinical & gene data

**DOI:** 10.3389/fonc.2024.1448966

**Published:** 2024-09-24

**Authors:** Jingru Li, Jingting Wang, Bangwei Cao

**Affiliations:** Department of Oncology, Beijing Friendship Hospital, Capital Medical University, Beijing, China

**Keywords:** non-small cell lung cancer, immune checkpoint inhibitor therapy, high-density lipoprotein, enrich analysis, machine learning, LMNA

## Abstract

**Objectives:**

Analyzing the impact of peripheral lipid levels on the efficacy of immune checkpoint inhibitor therapy in non-small cell lung cancer (NSCLC) patient populations and exploring whether it can serve as a biomarker for broadening precise selection of individuals benefiting from immunotherapy.

**Methods:**

We retrospectively collected clinical data from 201 cases of NSCLC patients receiving immune checkpoint inhibitor therapy. The clinical information included biochemical indicators like total cholesterol, triglycerides, high-density lipoprotein (HDL), and low-density lipoprotein (LDL). We utilized machine learning algorithms and Cox proportional hazards regression models to investigate independent predictors for both short-term and long-term efficacy of immunotherapy. Additionally, we concurrently developed a survival prediction model. Analyzing the Genes of Patients with Treatment Differences to Uncover Mechanisms

**Results:**

Correlation analysis revealed a significant positive association between HDL and ORR, DCR, and PFS. T-test results indicated that the high-HDL group exhibited higher DCR (81.97% vs. 45.57%) and ORR (61.48% vs. 16.46%). Kruskal-Wallis test showed that the high-HDL group had a longer median PFS (11 months vs. 6 months). Utilizing six machine learning algorithms, we constructed models to predict disease relief and stability. The model built using the random forest algorithm demonstrated superior performance, with AUC values of 0.858 and 0.802. Furthermore, both univariate and multivariate Cox analyses identified HDL and LDL as independent risk factors for predicting PFS. In patients with poor immunotherapy response, there is upregulation of BCL2L11, AKT1, and LMNA expression.

**Conclusion:**

HDL and LDL are independent factors influencing the survival prognosis of NSCLC patients undergoing immune checkpoint inhibitor therapy. HDL is expected to become new biomarkers for predicting the immunotherapy efficacy in patients with NSCLC. In patients with poor immunotherapy response, upregulation of the LMNA gene leads to apoptosis resistance and abnormal lipid metabolism.

## 1 Introduction

As programmed cell-death 1 (PD-1) and its ligand (PD-L1) targeted immunotherapy gradually becomes a predominant treatment modality, particularly for non-driver gene mutation patients, it significantly improves the survival rates of non-small cell lung cancer (NSCLC) patients. However, there remains a subset of individuals exhibiting poor treatment response to immune checkpoint inhibitor therapy. Currently, indicators such as Expression of PD-L1 protein (1–3), tumor mutation burden (TMB) (4) and microsatellite instability (MSI) (5) play a role in predicting the efficacy of immunotherapy. Nevertheless, these assessments have drawbacks such as high laboratory requirements and prolonged testing times, leading to limited clinical application. Peripheral blood samples, on the other hand, offer convenient sampling without invasive procedures, reflecting host immune status information, making them more accessible clinical indicators for predicting and dynamically monitoring the efficacy of immunotherapy in NSCLC.

Some studies suggest a connection between lipid metabolism alterations and the development of tumors. A cohort study indicates that NSCLC patients commonly exhibit blood lipid abnormalities, with a particularly close association between reduced levels of HDL and a higher risk of NSCLC (6). Research also indicates that compared to normal cells, tumor cells can synthesize more cholesterol not only to sustain their active proliferation but also to maintain the stability of the PD-L1 receptor expressed on tumor cells, which requires cholesterol (7, 8). Meanwhile, HDL and LDL primarily influence the occurrence and development of cancer through mechanisms such as modulating cholesterol transport and regulating inflammatory responses (9). Additionally, several clinical studies suggest the effectiveness of statin drugs in adjunct cancer treatment (10, 11). However, the value of blood lipid levels in the immune prognosis of NSCLC is not yet clear.

This study aims to explore the impact of blood lipid levels on the efficacy and prognosis of locally advanced and advanced NSCLC patients receiving immune checkpoint inhibitor therapy. It aims to construct a clinical prediction model to provide more refined screening indicators for the individualized treatment of NSCLC patients.

## 2 Material and methods

### 2.1 Patients

We retrospectively analyzed 201 advanced NSCLC patients treated with immune checkpoint inhibitor therapy at Beijing Friendship Hospital Cancer Center, Capital Medical University. All patients met the following criteria: 1. Pathologically confirmed diagnosis of non-small cell lung cancer; 2. Staged as III-IV according to the 8th edition TNM staging by the International Association for the Study of Lung Cancer, including untreated patients and those with recurrent disease after previous surgery; 3. Completed four or more cycles of monotherapy or combination therapy with anti-PD-1 or anti-PD-L1 monoclonal antibodies (mAb) and had clear efficacy assessment results; 4. Did not require additional lipid-lowering medications for diagnosed conditions during immunotherapy; 5. Eastern Cooperative Oncology Group (ECOG) score of 0-1; 6. No EGFR mutation, ALK translocation, or ROS1 fusion; 7. No immunosuppressive drug treatment required for other diseases. The study obtained approval from the Institutional Review Board, and due to the retrospective nature of the study, the need for informed consent was waived.

Clinical characteristics of all patients were collected, including age, gender, histology, prior lines of therapy, treatment type, tumor cell proportion score (TPS), Ki-67 protein level, liver metastasis, bone metastasis, brain metastasis, smoking history, diabetes history, statin drug usage history, body mass index (BMI), as well as fasting serum total cholesterol, serum triglycerides, high-density lipoprotein, and low-density lipoprotein within the first three natural days before the initial treatment. Patients were assessed for objective response rate (ORR), disease control rate (DCR), and progression-free survival (PFS). Tumor response was evaluated using Response Evaluation Criteria in Solid Tumors (RECIST) version 1.1. All patients underwent follow-up until death or data lock on August 1, 2023.

### 2.2 Statistical analysis

All data analysis and graphic plotting were conducted using R software version 4.1.2. ORR is defined as the ratio of the number of patients achieving complete response (CR) or partial response (PR) after four treatment cycles to the total number of patients. DCR is defined as the ratio of the number of patients achieving CR, PR, or stable disease (SD) after four treatment cycles to the total number of patients. PFS is defined as the time from the initiation of immune therapy to disease progression or death for any reason. Patients without events were censored at the last follow-up. Survival curves were estimated using the Kaplan-Meier method, and comparisons were made using the log-rank test. The chi-square test was employed to analyze differences in ORR and DCR between groups. Feature selection was performed using the random forest algorithm, followed by the construction of predictive models using k-nearest neighbors (KNN), backpropagation (BP) algorithm, support vector machine (SVM) algorithm, decision tree algorithm, random forest algorithm, and Xgboosting algorithm. The Cox regression model was utilized to investigate the correlation between various variables and the survival endpoint, incorporating factors with univariate p-values < 0.05 into multivariate analysis. Statistical tests were two-sided, and p-values < 0.05 were considered statistically significant.

### 2.3 Gene data resource and analysis

The datasets GSE136961 were downloaded from https://www.ncbi.nlm.nih.gov/geo/. GSEA were carried out to explore the biological functions in NSCLC patients receiving immunotherapy. Gene Ontology (GO) and Kyoto Encyclopedia of Genes and Genomes (KEGG) gene sets were obtained from the official GSEA website (https://www.gsea-msigdb.org/gsea/downloads.jsp).

## 3 Results

### 3.1 Demographic characteristics

**Figure 1:**
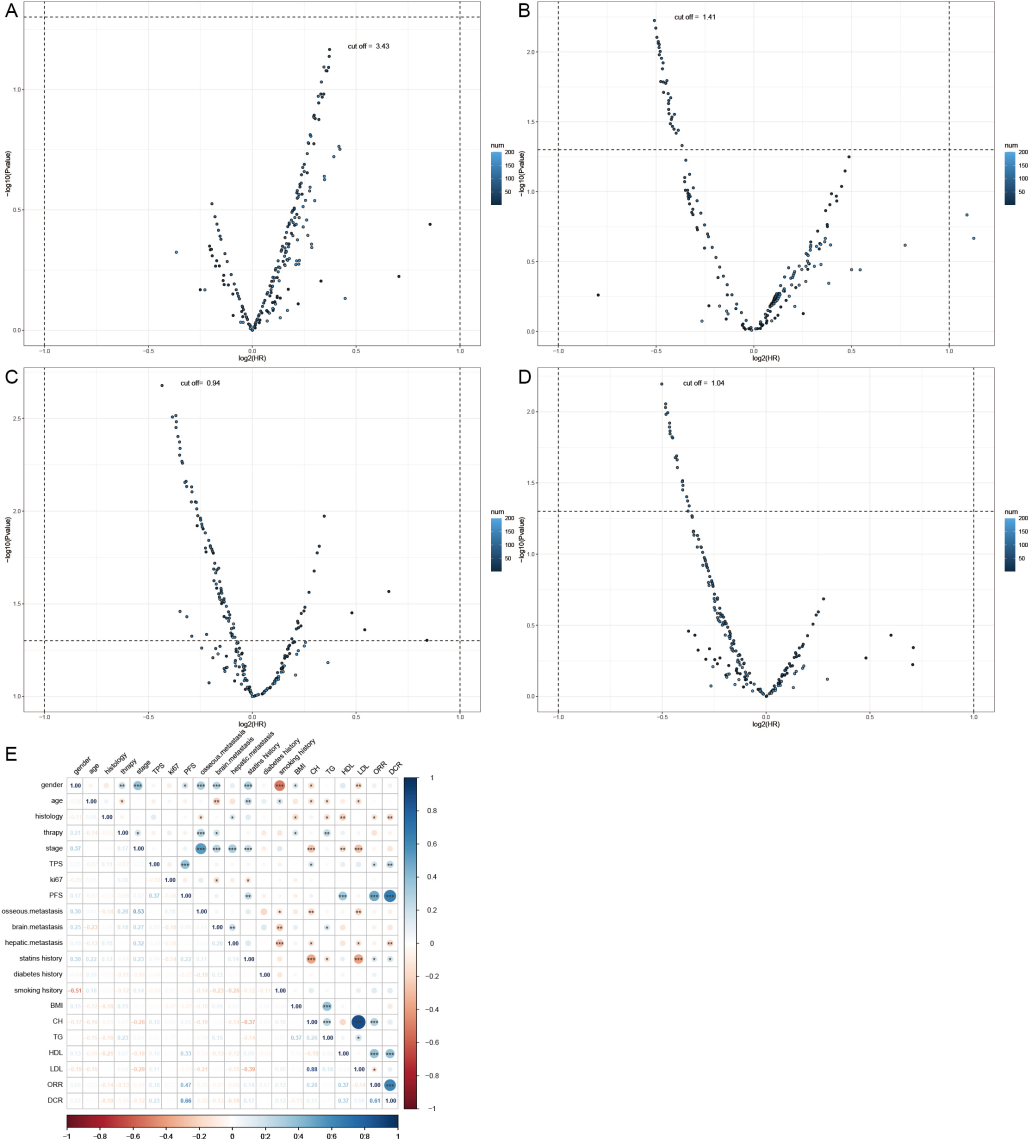
Demographic characteristics. **(A)** Calculating the optimal cutoff points using the iterative algorithm, for are 3.43 mmol/L, **(B)** for TG are 1.41 mmol/L, **(C)** for HDL are 0.94 mmol/L, **(D)** and for LDL are 1.04 mmol/L. **(E)** Calculate the correlation between demographic features and plot them as a bubble chart, bule indicates positive correlation, while red indicates negative correlation. PFS, progression-free survival; ORR, objective response rate; DCR, disease control rate *, p < 0.05; **, p < 0.01; ***, p < 0.001.

This study included a total of 201 patients (**Table 1**), with 136 males and 65 females. The age ranged from 38 to 82 years, with an average age of 65 years and a median age of 65 years. Among the 201 patients, adenocarcinoma accounted for the majority with 130 cases, followed by squamous cell carcinoma with 62 cases. Other histological types included 6 cases of non-small cell lung cancer with unclear histology, 2 cases of adenosquamous carcinoma, and 1 case of large cell carcinoma. Tumor PD-L1 expression was negative (TPS < 50%) in 155 patients and positive (TPS ≥ 50%) in 46 patients. Among the patients who received immune therapy, 57 had a regular history of statin drug usage before immunotherapy. Due to differences in blood lipid levels between tumor patients and the normal population, normal indicators were not selected as grouping criteria. In this study, we employed an iterative algorithm to establish a survival function related to progression-free survival (PFS). We determined the optimal cutoff points for cholesterol (CH), triglycerides (TG), high-density lipoprotein (HDL), and low-density lipoprotein (LDL) based on the maximum statistical differences (**Figures 1A–D**), dividing the patients into high-level and low-level groups. The optimal cutoff values were 3.43 mmol/L for CH, 1.41 mmol/L for TG, 0.94 mmol/L for HDL, and 1.04 mmol/L for LDL.

**Table 1:** Baseline demographics.

characteristics	Overall	characteristics	Overall
	(N=201)		(N=201)
gender		smoke history	
male	136 (67.7%)	yes	116 (57.7%)
female	65 (32.3%)	no	85 (42.3%)
age(years)		history of diabetes	
Mean (SD)	65.24 (7.32)	yes	77 (38.3%)
Median [Min, Max]	65 [38.0, 82.0]	no	124 (61.7%)
treatment line		osseous metastasis	
firstline	152 (75.6%)	yes	67 (33.3%)
secondline	31 (15.4%)	no	134 (66.7%)
≥thirdline	18 (9.0%)	brain metastasis	
stage		yes	23 (11.4%)
III	73 (36.3%)	no	178 (88.6%)
IV	128 (63.7%)	hepatic metastasis	
treatment type		yes	30 (14.9%)
monotherapy	21 (10.4%)	no	171 (85.1%)
combination therapy	180 (89.6%)	cholesterol	
TPS		low	58 (28.9%)
<50%	155 (77.1%)	high	143 (71.1%)
≥50%	46 (22.9%)	triglyceride	
ki67		low	88 (43.8%)
<25%	42 (20.9%)	high	113 (56.2%)
≥25%	159 (79.1%)	high-density lipoprotein	
histology		low	79 (39.3%)
adenocarcinoma	130 (64.7%)	high	122 (60.7%)
squamous cell carcinoma	62 (30.8%)	low-density lipoprotein	
other	9 (4.5%)	low	113 (56.2%)
PFS(mouths)		high	88 (43.8%)
Mean (SD)	10.3 (7.08)	BMI	
Median [Min, Max]	9.00 [3.00, 37.0]	≤23.8	109(54.23%)
therapeutic effect evaluation		>23.8	92 (45.77%)
PR	88 (43.8%)	statins history	
SD	48 (23.9%)	yes	57 (28.4%)
PD	65 (32.3%)	no	144 (71.6%)

PR, partial response; SD, stable disease; PD, progressive disease.

Correlation analyses of baseline data were conducted (**Figure 1E**), revealing a positive correlation of HDL levels with objective response rate (ORR) (0.37), disease control rate (DCR) (0.37), and PFS (0.33). Cholesterol levels also showed a positive correlation with ORR (0.28). Similarly, the history of statin drug usage was a positive factor for ORR (0.14), DCR (0.17), and PFS (0.22). LDL levels were a negative factor for ORR (-0.14). Additionally, a strong positive correlation (0.88) was observed between CH levels and LDL levels. Smoking history showed a strong correlation with male patients (0.51), while female patients were more prone to brain (0.25) and bone metastasis (0.3), potentially associated with longer PFS (0.17).

### 3.2 Short-term treatment response

After the fourth treatment cycle, we evaluated target lesions in patients according to RECIST1.1 criteria and calculated ORR and DCR for each group separately (**Table 2**). We used Chi-square tests to analyze differences between groups, applying Fisher’s test when n<5. The results show that the high-cholesterol group achieved a superior ORR (51.05% vs. 25.86%). The low triglyceride group exhibited higher DCR (78.41% vs. 59.29%) and ORR (53.41% vs. 36.28%) compared to the high-level group. Similarly, the high HDL group significantly improved both DCR (81.97% vs. 45.57%) and ORR (61.48% vs. 16.46%), while LDL showed no significant impact on DCR and ORR. The group with a history of statin drug usage had a higher DCR (80.77% vs. 63.09%) compared to the group without a history, but it had no significant impact on ORR. Consistent with other studies, higher TPS indicated a higher treatment response rate, and younger individuals had better DCR and ORR. Additionally, patients without liver metastasis had a higher DCR (71.35% vs. 46.67%) than the group with liver metastasis.

**Table 2:** Impact of clinical characteristics on DCR and ORR.

Characteristics	DCR	P-value	ORR	P-value	Characteristics	DCR	P-value	ORR	P-value
**gender**					**CH**				
male	65.44%	0.417	41.18%	0.355	low	67.24%	1	25.86%	0.00191
female	72.31%		49.23%		high	67.83%		51.05%	
**age**					**TG**				
≤65	76.83%	0.0313	54.88%	0.0129	low	78.41%	0.0065	53.41%	0.0223
>65	61.34%		36.13%		high	59.29%		36.28%	
**treatment line**					**HDL**				
fistline	68.42%	0.496	46.71%	0.137	low	45.57%	<0.001	16.46%	<0.001
secondline	70.97%		41.94%		high	81.97%		61.48%	
≥thirdline	55.56%		22.22%		**LDL**				
**treatment type**					low	57.89%	0.99	40.71%	0.394
monotherapy	71.43%	0.886	42.86%	1	high	67.05%		47.73%	
combination	67.22%		43.89%		**smoking history**				
**stage**					yes	61.18%	0.126	49.14%	0.1
III	75.34%	0.109	46.58%	0.649	no	72.41%		36.47%	
IV	63.28%		42.19%		**diabetes history**				
**TPS**					yes	67.74%	1	41.13%	0.415
<50%	60.65%	<0.001	38.06%	0.0047	no	67.53%		48.05%	
≥50%	91.30%		63.04%		**statins history**				
**ki67**					yes	80.77%	0.0296	55.77%	0.0627
≤25%	64.29%	0.734	42.86%	1	no	63.09%		39.60%	
≥25%	67.92%		44.03%		**osseous metastasis**				
**histology**					yes	62.69%	0.365	40.30%	0.580
LUAD	70.50%	0.402	46.04%	0.35	no	70.15%		45.52%	
LUSC	62.26%		41.51%		**brain metastasis**				
other	55.56%		22.22%		yes	52.17%	0.147	34.78%	0.483
**BMI**					no	69.66%		44.94%	
≤23.5	68.87%	0.642	45.03%	0.647	**hepatic metastasis**				
>23.5	64.00%		40.00%		yes	46.67%	0.0141	36.67%	0.514
					no	71.35%		45.03%	

Combination, combination therapy; LUAD, adenocarcinoma of the lung; LUSC, squamous cell carcinoma of the lung.

**Figure 2:**
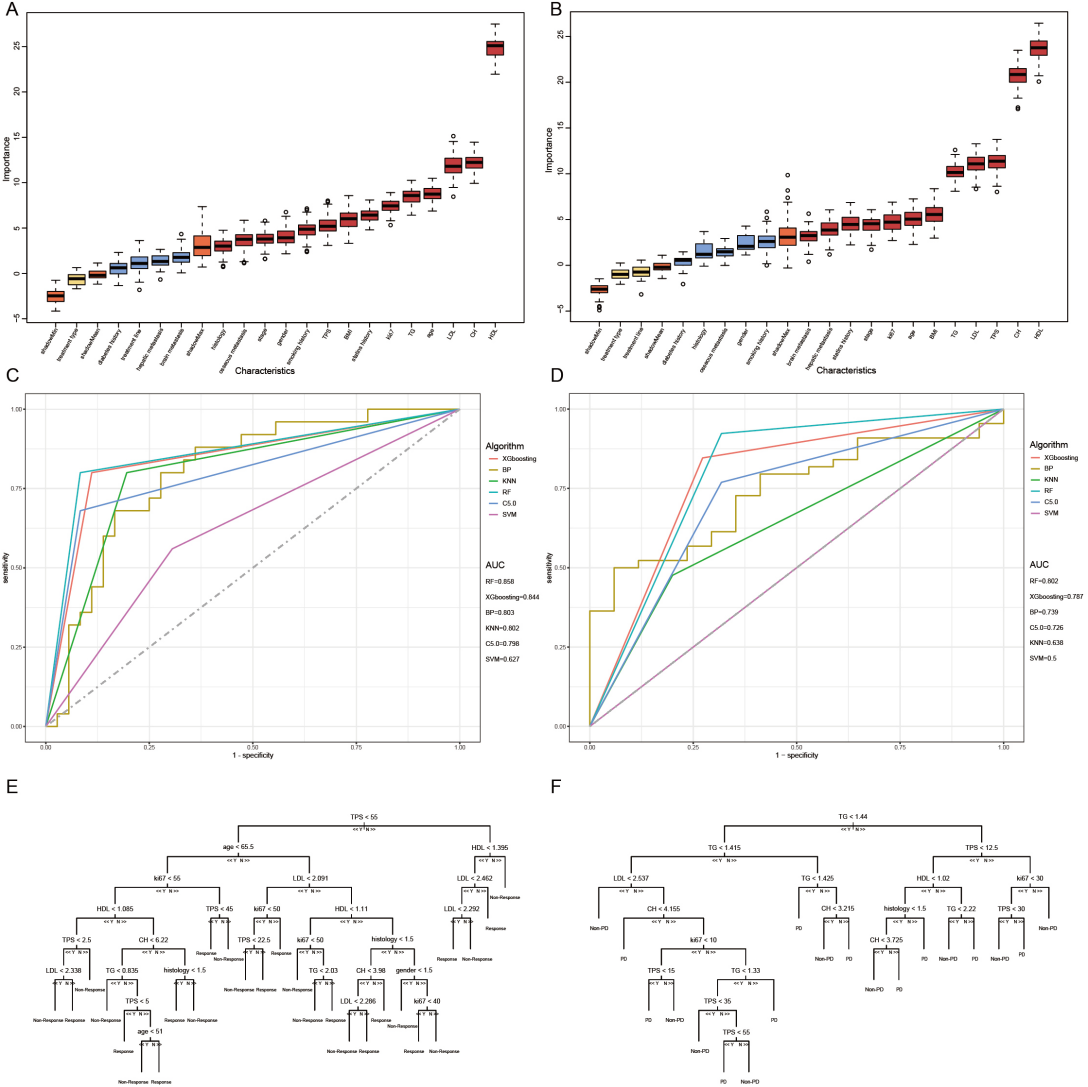
Short-term efficacy prediction model. **(A)** We employed the Boruta algorithm to calculate the importance of each feature in influencing disease remission, ranking them from low to high. Red indicates that the feature significantly affects remission, blue suggests some impact but lacks statistical significance, and yellow denotes no influence. HDL was identified as the most influential factor on disease response, **(B)** while both HDL and CH had the greatest impact on preventing disease progression. **(C)** We plotted ROC curves for six machine learning models, and the random forest algorithm exhibited the best performance in predicting disease response, with an AUC of 0.858. **(D)** In the model predicting disease progression, the random forest algorithm demonstrated the best performance, achieving an AUC of 0.802. **(E)** Flowchart for predicting whether patients can achieve disease response after four cycles of treatment. **(F)** Flowchart for predicting whether patients can prevent disease progression after four cycles of treatment.

DCR and ORR, as short-term indicators for immune therapy assessment, demonstrated correlation with blood lipid levels. Therefore, we constructed corresponding predictive models using machine learning algorithms. Initially, the Boruta algorithm was employed for feature selection, identifying significant factors for ORR (**Figure 2A**): HDL, CH, LDL, age, TG, Ki-67, statins history, BMI, TPS, smoking history, gender, stage, osseous metastasis, histology. Similarly, significant factors for DCR (**Figure 2B**) included HDL, CH, TPS, LDL, TG, BMI, age, Ki-67, stage, hepatic metastasis, brain metastasis. After feature selection, patients were divided into training and validation sets in a 7:3 ratio, and predictive models were constructed using random forest, BP algorithm, SVM algorithm, decision tree algorithm, Xgboosting algorithm, and KNN algorithm, and the performance of each model was evaluated (**Figures 2C, D**). The random forest model demonstrated the best performance in predicting ORR (**Figure 2E**), with an AUC of 0.858, Kappa of 0.726, and F1 of 0.833 (**Table 3**). In the prediction model for DCR, the random forest model also performed optimally (**Figure 2F**), achieving an AUC of 0.802, Kappa of 0.630, and F1 of 0.878 (**Table 3**).

**Table 3:** Model Evaluation.

	Predictive model for disease response					Predictive model for disease progression-free				
	Accuracy(95% CI)	Sensitivity	Specificity	Kappa	F1	Accuracy(95% CI)	Sensitivity	Specificity	Kappa	F1
KNN	0.803(0.682, 0.894)	0.804	0.800	0.533	0.886	0.689(0.557, 0.801)	0.476	0.800	0.403	0.771
C5.0	0.820(0.701, 0.906)	0.917	0.680	0.616	0.756	0.738(0.609, 0.842)	0.769	0.682	0.442	0.789
BP	0.754(0.627, 0.855)	0.806	0.680	0.489	0.694	0.787(0.663, 0.881)	0.900	0.571	0.501	0.842
SVM	0.639(0.506, 0.758)	0.694	0.560	0.254	0.56	0.656(0.523, 0.773)	0.000	1.000	0	–
RF	0.869(0.756, 0.942)	0.917	0.800	0.726	0.833	0.836(0.719, 0.919)	0.923	0.682	0.630	0.878
Xgboosting	0.853(0.738, 0.930)	0.889	0.800	0.693	0.816	0.803(0.682, 0.894)	0.846	0.727	0.573	0.833

CI, confidence interval; F1, F1 Score.

We can conclude that the blood lipid levels of patients have a certain impact on the efficacy of immune therapy, especially HDL, which provides indications for short-term disease relief and stability. Using blood lipid level data can also construct excellent efficacy prediction models.

### 3.3 Long-term treatment response

To elucidate the long-term impact of blood lipid levels on the efficacy of immune therapy, we first analyzed the differences in median PFS under clinical feature grouping using Kruskal-Wallis test. The results showed (**Table 4**) that the high-HDL group (11 months vs. 6 months), high-TPS group (15 months vs. 8 months), low-TG group (11 months vs. 8 months), low-LDL group (10 months vs. 9 months), and statin drug usage history group (12 months vs. 8 months) had longer PFS.

**Table 4:** Impact of Clinical Characteristics on PFS.

Characteristics	median PFS(mos)	P-value	Characteristics	median PFS(mos)	P-value
**gender**			**CH**		
male	9	0.125	low	8	0.623
female	10		high	10	
**age(years)**			**TG**		
≤65	9	0.445	low	11	0.004
>65	10		high	8	
**treatment line**			**HDL**		
fistline	10	0.515	low	6	<0.001
secondline	8		high	11	
≥thirdline	8		**LDL**		
**treatment type**			low	10	0.049
monotherapy	10	0.540	high	9	
combination	9		**smoking history**		
**stage**			yes	10	0.959
III	10	0.968	no	8	
IV	8		**diabetes history**		
**TPS**			yes	10	0.935
<50%	8	<0.001	no	9	
≥50%	15		**statins history**		
**ki67**			yes	12	0.003
≤25%	10	0.664	no	8	
≥25%	9		**osseous metastasis**		
**histology**			yes	7	0.370
LUAD	10	0.675	no	10	
LUSC	8		**brain metastasis**		
other	5		yes	6	0.884
**BMI**			no	9.5	
≤23.5	9	0.877	**hepatic metastasis**		
>23.5	9.5		yes	5	0.516
			no	10	

PFS, progression-free survival; Combination, combination therapy; LUAD, adenocarcinoma of the lung; LUSC, squamous cell carcinoma of the lung,mos, months.

**Figure 3:**
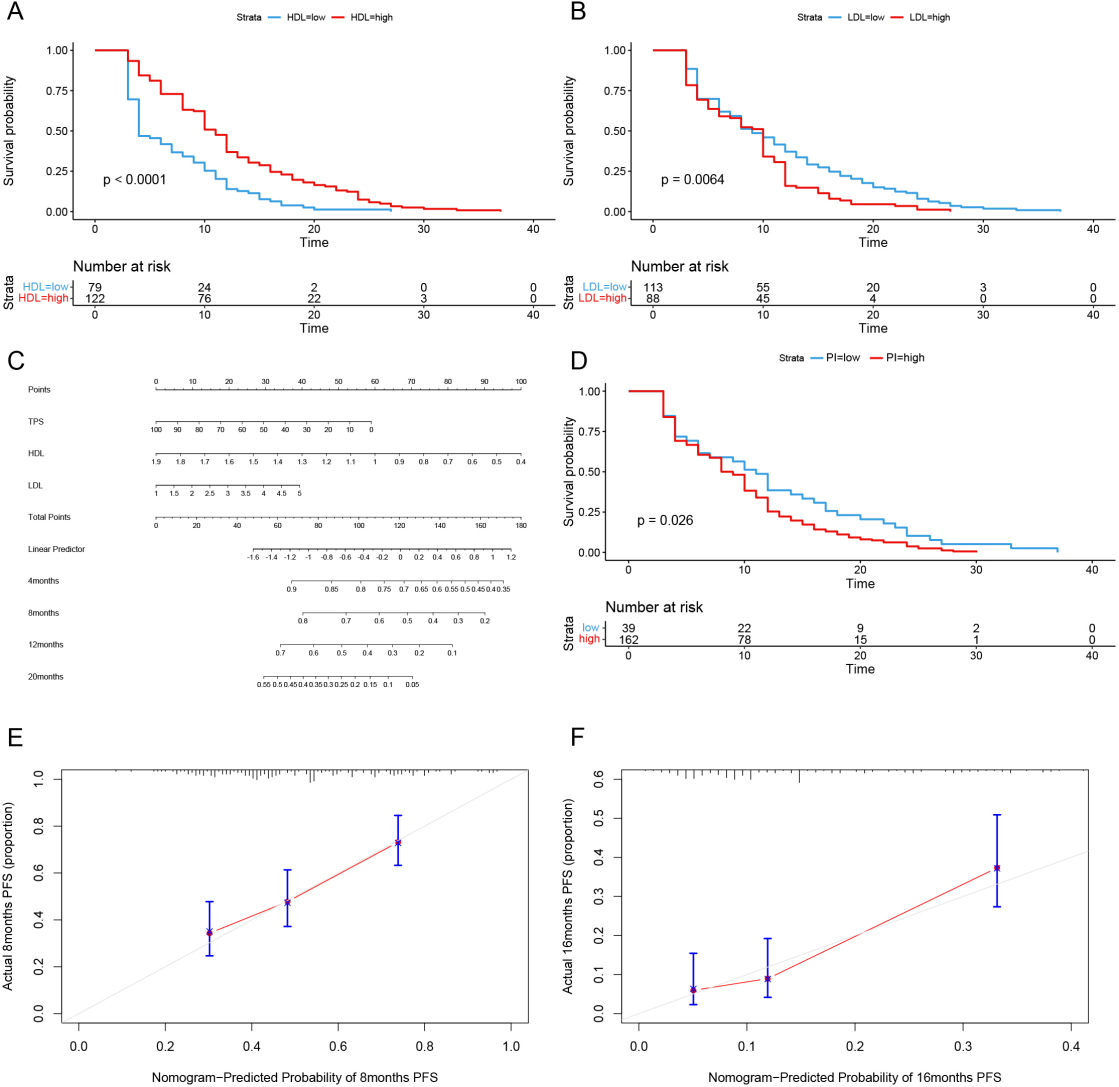
Long-term efficacy prediction model. **(A)** After Univariate and multivariate Cox regression analysis, HDL was identified as an independent risk factor for predicting progression-free survival (PFS). Grouped by cutoff points (0.94 mmol/L), Kaplan-Meier curves were plotted, revealing a longer survival period in the high-level group compared to the low-level group (p < 0.0001). **(B)** After Univariate and multivariate Cox regression analysis, LDL was identified as an independent risk factor for predicting progression-free survival (PFS). Grouped by cutoff points(1.04 mmol/L), Kaplan-Meier curves were plotted, revealing a longer survival period in the high-level group compared to the low-level group (p < 0.0064). **(C)** Based on the multivariate Cox regression analysis, a Nomogram plot was generated. **(D)** Grouped by cutoff points (0.2004558) of PI, Kaplan-Meier curves were plotted, revealing a longer survival period in the low PI compared to the high PI group (p=0.026). **(E)** The calibration curve of the model predicting at the 8th month. **(F)** The calibration curve of the model predicting at the 16th month.

Subsequently, we conducted univariate COX analysis based on PFS for clinical features (**Table 5**). Factors with statistically significant (P<0.05) and clinically meaningful results, such as HDL, LDL, TG, TPS, and statin drug usage history, were included in the multivariate Cox proportional hazards regression model analysis (**Table 5**). The results indicated that TPS, HDL levels, and LDL levels were independent prognostic factors for PFS in patients with locally advanced and advanced NSCLC undergoing immune therapy (**Figures 3A, B**).

**Table 5:** Univariate and multivariate Cox-regression analysis of the PFS.

	Univariate			Multivariate		
Characteristics	HR	95% CI	P-value	HR	95% CI	P-value
TPS						
≥50% vs. <50%	0.46	0.46-2.18	<0.001	0.48	0.18-4.10	<0.001
statins history						
yes vs. no	0.65	0.65-1.55	0.007	0.9	0.19-0.53	0.59
TG						
high vs. low	1.47	0.68-1.47	0.008	0.15	0.16-1.17	0.34
HDL						
high vs. low	0.45	0.45-2.22	<0.001	0.53	0.16-4.12	<0.001
LDL						
high vs. low	1.52	0.66-1.52	0.005	1.56	0.16-2.82	0.005
gender						
male vs. female	0.74	0.74-1.35	0.053	–	–	–
age(years)						
>65 vs. ≤65	0.92	0.92-1.08	0.588	–	–	–
histology						
adenocarcinoma vs.other	0.85	0.85-1.17	0.3	–	–	–
treatment line						
firstline vs.non-firstline	0.88	0.88-1.14	0.517	–	–	–
treatment type						
monotherapy vs.other	0.88	0.88-1.14	0.517	–	–	–
stage						
III vs.IV	0.82	0.82-1.22	0.187	–	–	–
ki67						
<25% vs. ≥25%	1.22	0.82-1.22	0.266	–	–	–
osseou metastasis						
yes vs. no	1.02	0.98-1.02	0.897	–	–	–
brain metastasis						
yes vs. no	1.29	0.77-1.29	0.258	–	–	–
hepatic metastasis						
yes vs. no	1.12	0.89-1.12	0.57	–	–	–
diabetes history						
yes vs. no	1.19	0.84-1.19	0.231	–	–	–
smoking history						
yes vs. no	1.1	0.91-1.1	0.514	–	–	–
BMI						
>23.8 vs. ≤23.8	0.98	0.98-1.02	0.897	–	–	–
CH						
high vs. low	0.75	0.75-1.32	0.079	–	–	–

CI, confidence interval; HR, hazard ratio; PFS, progression-free survival.

Based on the multivariate Cox regression model, probability prediction line charts for PFS at 4 months, 12 months, and 20 months were plotted (**Figure 3C**). The total score was obtained by adding the scores corresponding to each influencing factor, allowing estimation of PFS probabilities for patients at 4, 8, 12, and 20 months. The C-index of model is 0.71, and calibration curves for PFS probability prediction models at 8 months and 16 months were plotted (**Figures 3E, F**). According to the model formula, patients’ prognostic index (PI) was calculated, and the population was divided into high-risk and low-risk groups based on the optimal cutoff value (0.2004558) for PI for survival analysis (**Figure 3D**). The results showed a statistically significant difference (P=0.026), confirming that this model can significantly differentiate the survival benefits of NSCLC patients clinically receiving immune therapy.

### 3.4 Exploration of gene mechanisms

**Figure 4:**
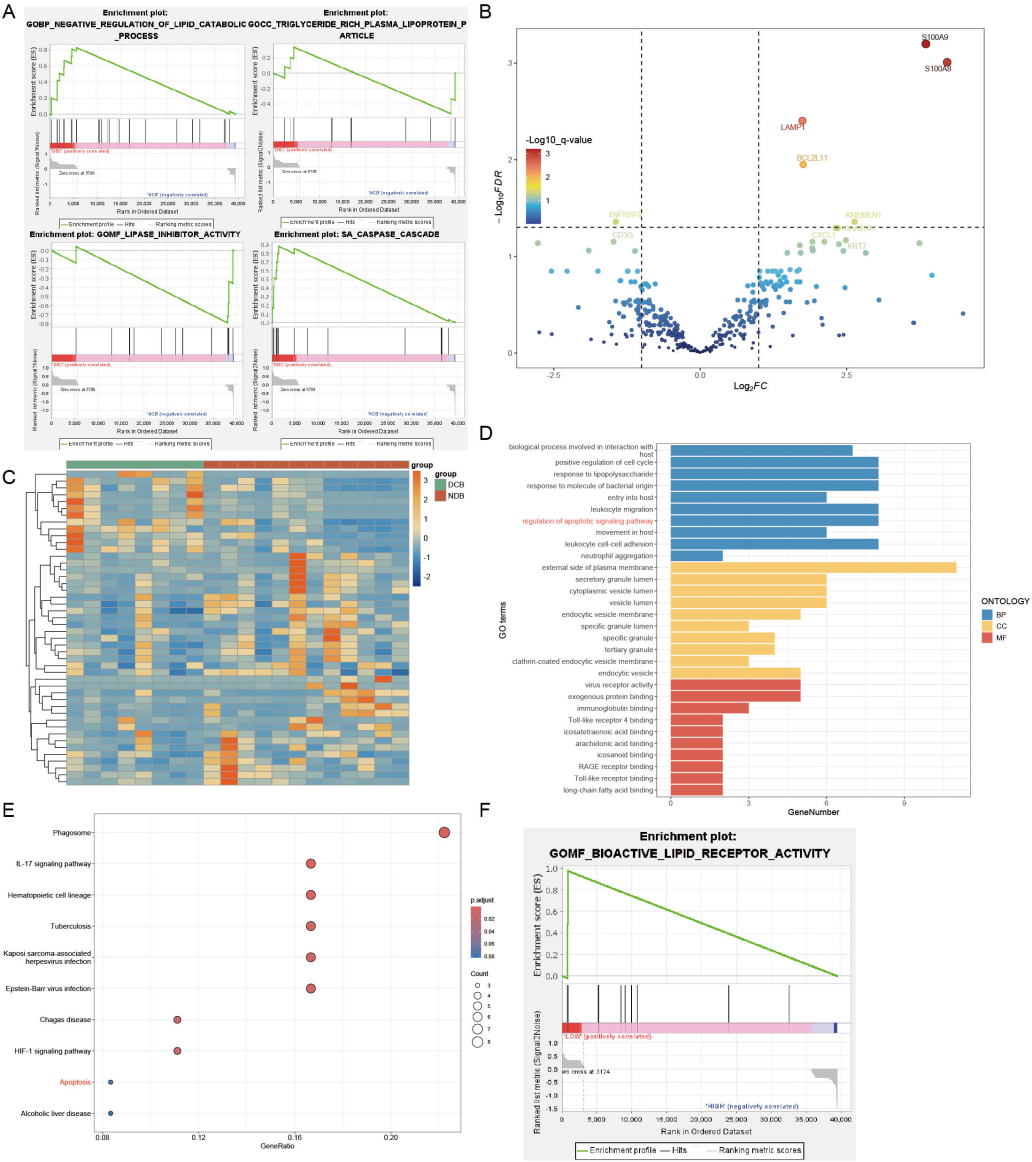
Genesets analysis. **(A)** DCB group enriched in the NEGATIVE REGULATION OF LIPID CATABOLIC PROCESS and CASPASE CASCADE. NDB group enriched in the LIPASE INHIBITOR ACTIVITY and TRIGLYCERIDE RICH PLASMA LIPOPROTEIN PARTICLE. **(B)** Part of the differentially expressed genes were visualized in a volcano plot. **(C)** Differentially expressed genes were visualized in a heatmap. **(D)** GO enrichment analysis of differentially expressed genes. **(E)** Kegg enrichment analysis of differentially expressed genes. **(F)** GSEA analysis: the high LMNA expression group enriched in the BIOACTIVE LIPID RECEPTOR ACTIVITY.

We observed that reduced HDL levels in NSCLC patients might be associated with poor response to immunotherapy. To elucidate the mechanisms underlying this clinical phenomenon, we conducted further research. Initially, we obtained data from the GSE136961 dataset in the GEO database, which includes data from 10 patients regularly undergoing immunotherapy. Based on their response to immunotherapy, patients were divided into the Durable Clinical Benefit (DCB) group and the Non-Durable Benefit (NDB) group. Gene Set Enrichment Analysis (GSEA) comparing gene functions between these two groups (**Figure 4A**) revealed that genes in the NDB group were significantly enriched in the LIPASE INHIBITOR ACTIVITY and TRIGLYCERIDE RICH PLASMA LIPOPROTEIN PARTICLE, whereas genes in the DCB group were enriched in the NEGATIVE REGULATION OF LIPID CATABOLIC PROCESS. This suggests that the poor response to immunotherapy in NSCLC patients may be associated with upregulation of genes such as LPL and the ANX family, leading to dysregulation of lipid metabolism and consequently reduced HDL levels.

Furthermore, we observed silencing of the CASPASE CASCADE in the NDB group (**Figure 4A**), indicating potential resistance to apoptosis in these tumor cells, which may contribute to impaired immunotherapy efficacy. To investigate further, we analyzed the GSE136961 dataset, which includes expression information of 395 immune-related genes from 20 patients, obtained through the Oncomine Immune Response Research Assay. Using the R package DESeq2, we assessed gene expression differences between the two groups (**Figures 4B, C**) and identified 46 differentially expressed genes (**Table 6**), with 12 genes downregulated and 34 genes upregulated.

**Table 6:** Differential Genes: DCB vs NDB.

genename	baseMean	log2FoldChange	lfcSE	stat	pvalue	padj
NOS2	53.367	23.998	2.153	11.148	<0.001	<0.001
MAGEA4	20.705	22.674	3.033	7.475	<0.001	<0.001
HLA-B	5788.687	-6.411	0.984	-6.517	<0.001	<0.001
S100A9	20928.990	3.853	0.855	4.509	<0.001	<0.001
S100A8	46760.396	4.217	0.966	4.366	<0.001	<0.001
LAMP1	3610.221	1.745	0.435	4.008	<0.001	0.004
BCL2L11	1986.983	1.758	0.473	3.717	<0.001	0.011
TNFRSF9	671.027	-1.441	0.438	-3.291	0.001	0.044
KREMEN1	3283.787	2.635	0.802	3.287	0.001	0.044
CXCL1	5996.311	2.316	0.727	3.186	0.001	0.051
KIAA0101	1512.716	2.348	0.737	3.186	0.001	0.051
KRT7	28461.632	2.486	0.809	3.075	0.002	0.068
CD3G	1920.498	-1.479	0.490	-3.016	0.003	0.070
CXCL8	10091.901	2.119	0.704	3.008	0.003	0.070
CDK1	3692.393	1.917	0.639	2.997	0.003	0.070
MS4A1	229.253	-2.772	0.940	-2.948	0.003	0.073
VTCN1	219.573	3.742	1.267	2.954	0.003	0.073
FCGR3B	799.756	2.368	0.810	2.923	0.003	0.075
BRCA1	899.447	1.701	0.587	2.897	0.004	0.077
LRG1	828.227	1.915	0.669	2.860	0.004	0.082
TFRC	35738.570	1.923	0.684	2.812	0.005	0.087
CD22	453.002	-1.902	0.674	-2.824	0.005	0.087
PTGS2	3476.746	2.445	0.878	2.784	0.005	0.088
CD37	2187.025	-1.115	0.401	-2.780	0.005	0.088
NOTCH3	30451.709	1.716	0.629	2.727	0.006	0.092
LCN2	43452.412	2.827	1.034	2.734	0.006	0.092
TUBB	18790.453	1.488	0.543	2.740	0.006	0.092
RB1	3733.450	1.534	0.595	2.577	0.010	0.137
MAD2L1	1888.693	1.692	0.659	2.567	0.010	0.137
G6PD	6505.701	1.377	0.559	2.463	0.014	0.142
AKT1	11206.685	1.153	0.474	2.432	0.015	0.142
CD53	174.898	-2.540	1.034	-2.455	0.014	0.142
NECTIN2	1367.132	1.287	0.529	2.433	0.015	0.142
GPR18	128.028	-1.746	0.716	-2.440	0.015	0.142
CXCR5	156.531	-2.274	0.930	-2.445	0.014	0.142
HLA-DOA	846.120	-1.071	0.435	-2.464	0.014	0.142
ITGAE	758.644	1.233	0.495	2.491	0.013	0.142
M6PR	16586.141	1.015	0.413	2.457	0.014	0.142
MRC1	6065.714	-1.235	0.497	-2.487	0.013	0.142
IKZF3	1730.831	-1.240	0.509	-2.433	0.015	0.142
MELK	2893.216	1.655	0.660	2.506	0.012	0.142
RPS6	112311.722	1.333	0.558	2.389	0.017	0.156
KRT5	413503.456	3.956	1.663	2.378	0.017	0.157
PTPN11	9593.958	1.446	0.614	2.354	0.019	0.164
TNFRSF18	1210.996	1.430	0.612	2.335	0.020	0.165
LMNA	28475.663	1.009	0.432	2.337	0.019	0.165

Gene Ontology (GO) and Kyoto Encyclopedia of Genes and Genomes (Kegg) enrichment analyses of these 46 genes (**Figures 4D, E**) showed that S100A9, S100A8, BCL2L11, BRCA1, PTGS2, RB1, AKT1, and LMNA were enriched in the regulation of apoptotic signaling pathway function, while BCL2L11, AKT1, and LMNA were significantly enriched in the Apoptosis pathway. These results indicate that upregulation of BCL2L11, AKT1, and LMNA in the NDB group may collectively point to a function of inhibiting apoptosis, which could be a key factor in the poor response of these patients to immunotherapy.

To further investigate the relationship between tumor cell apoptosis resistance and HDL levels, we divided the patients in the GSE136961 dataset into high-expression and low-expression groups based on LMNA expression levels. GSEA analysis revealed that the high LMNA expression group showed significant resistance to the BIOACTIVE LIPID RECEPTOR ACTIVITY (**Figure 4F**).

Therefore, when tumor cells resist apoptosis signals by upregulating BCL2L11, AKT1, and LMNA genes, they simultaneously exhibit reduced responsiveness to lipid receptor activity. This change subsequently affects the concentration of HDL in the plasma. We believe that this mechanism may explain the observed reduction in HDL levels in patients with high LMNA expression, or in other words, NSCLC patients with poor immune therapy outcomes.

## 4 Discussion

In this study, we collected blood lipid data from patients with non-small cell lung cancer (NSCLC) before undergoing immunotherapy and analyzed its relationship with treatment efficacy. High-density lipoprotein (HDL) stood out, demonstrating remarkable effectiveness in promoting disease remission and long-term stability. We constructed predictive models for short-term disease relief and disease stability, with blood lipid data playing a crucial role. This confirms the potential of HDL as a novel biomarker for predicting the efficacy of immunotherapy in patients with advanced NSCLC. Additionally, we explored the potential mechanisms behind these two phenomena. The LMNA gene appears to link the two, assisting tumor cells in resisting apoptosis while simultaneously impeding the process of lipid transfer from the tumor cells to the outside.

After four cycles of treatment, patients with low HDL levels lacked responsiveness to immunotherapy, with lower DCR, ORR, and PFS compared to patients with normal HDL levels. Although HDL may not directly participate in the tumor cells’ response to the immune system, our study suggests that it can act as an indicator, with the LMNA gene being the culprit behind this phenomenon. Our research found that patients with poor immunotherapy outcomes generally exhibited upregulation of the BCL2L11, AKT1, and LMNA genes. BCL2L11 and AKT1 have been confirmed in multiple studies to resist apoptotic signals, serving as key players in tumor cells’ defense against the immune system (12, 13). The role of the lamin A/C protein encoded by the LMNA gene in the apoptosis process remains under investigation. Previous research suggests that as a major component of the nuclear lamina, lamin A/C is targeted during apoptosis. However, some studies propose that tumor cells may resist the transmission and activation of apoptotic signals within the nucleus by increasing the content of nuclear lamina proteins (14).

Although previous studies have indicated a significant role of cholesterol (CH) in the development of lung cancer, our research found its correlation with the efficacy of immunotherapy to be less significant. This may be attributed to its dual role. For instance, increasing intracellular free cholesterol can enhance the effector functions and proliferation of CD8^+^ T cells (15), but, conversely, the elevated cholesterol concentration in the tumor microenvironment can lead to the exhaustion of CD8^+^ T cells (16). Accumulation of cholesterol in macrophages can halt the apoptosis of tumor-infiltrating macrophages, preventing the transformation from M2 to M1 macrophages. Knocking out the ABCG1 gene responsible for this transport pathway in mice resulted in excellent anti-tumor characteristics (17). Moreover, lipid rafts primarily composed of cholesterol are obligatory for multiple signaling pathways involved in cancer occurrence and development (18). The contradictory nature of these effects hinders serum cholesterol from becoming a robust predictive indicator for immunotherapy efficacy in non-small cell lung cancer.

Although low-density lipoprotein (LDL) and triglycerides (TG) did not exhibit as pronounced predictive capabilities as HDL, they still held certain value. LDL has been found to regulate tumor growth and migration through signaling pathways in various cancers, and LDL receptor (LDLR) expression is upregulated in many cancers, primarily directing toward the significant increase in cholesterol demand due to metabolic reprogramming in tumor cells (19–21). Some studies have indicated that TG can lead to the dysfunction of the immune surveillance system composed of natural killer (NK) cells (22). Additionally, in lung cancer patients, a significant decrease in peripheral blood dendritic cell (DC) count and intracellular lipid accumulation, mainly TG, leads to reduced DC antigen presentation function (23). However, it is essential to note that LDL and TG may not be as prominent in prediction because LDL levels are often influenced by fluctuations in other lipid levels, compromising their independence. Moreover, the role of TG may be more accentuated in cardiovascular diseases, interfering with the prediction of tumor treatment efficacy.

In recent years, statin drugs have gained recognition for their ability to combat tumors, showing excellent anti-cancer performance in both *in vitro* and clinical trials (24, 25). Statins not only inhibit abnormal cholesterol uptake and synthesis by tumor cells but also promote tumor cell apoptosis through the mechanism mediated by geranylgeranyl pyrophosphate (GGPP) (26). Furthermore, statins can inhibit tumor metastasis by disrupting the geranylgeranylation and farnesylation of small GTPases (27). However, in this study, we only observed a clear positive impact of statin drugs on short-term efficacy, and their status as independent prognostic factors for long-term survival was not confirmed. This could be attributed to the limitations of our study, including a retrospective nature and a relatively small sample size. Some patients had follow-up periods exceeding one year, during which detailed collection of drug dosage adjustments or discontinuations was not conducted.

Therefore, our findings align with the perspective that blood lipid levels can serve as valuable indicators for predicting the efficacy of immune checkpoint inhibitor therapy. HDL levels reflect the expression of the LMNA gene in tumor tissues, which in turn indicates the extent of apoptosis resistance in tumor cells. Additionally, we developed predictive models using machine learning algorithms, demonstrating the feasibility of incorporating blood lipid levels into clinical prediction models. We explored the impact of blood lipid levels on long-term treatment response, revealing that groups with high HDL, low LDL, and a history of statin use have a longer progression-free survival (PFS). Multivariate Cox regression analysis identified tumor proportion score (TPS), HDL levels, and LDL levels as independent prognostic factors for PFS. The constructed predictive model showed significant discriminative power in categorizing patients into high-risk and low-risk groups.

However, due to the retrospective nature of this study and the relatively small sample size, these conclusions lack generalizability. Additionally, the absence of a validation cohort necessitates external validation in larger cohorts to confirm the robustness of the predictive models. The underlying mechanisms of the observed correlations between blood lipid levels and treatment response require further *in vivo* and *in vitro* investigation.

## Data Availability

The raw data supporting the conclusions of this article will be made available by the authors, without undue reservation.
