# Corrigendum: Exploring the impact of HDL and LMNA gene expression on immunotherapy outcomes in NSCLC: a comprehensive analysis using clinical & gene data

**DOI:** 10.3389/fonc.2025.1575160

**Published:** 2025-02-25

**Authors:** Jingru Li, Jingting Wang, Bangwei Cao

**Affiliations:** Department of Oncology, Beijing Friendship Hospital, Capital Medical University, Beijing, China

**Keywords:** non-small cell lung cancer, immune checkpoint inhibitor therapy, high-density lipoprotein, enrich analysis, machine learning, LMNA

In the published article, an author name was incorrectly written as Banwei Cao. The correct spelling is Bangwei Cao.

The authors apologize for this error and state that this does not change the scientific conclusions of the article in any way. The original article has been updated.

